# Draft Genomic Analysis of an Avian Multidrug Resistant *Morganella morganii* Isolate Carrying *qnrD1*

**DOI:** 10.3389/fmicb.2016.01660

**Published:** 2016-10-25

**Authors:** Daniela Jones-Dias, Lurdes Clemente, Inês B. Moura, Daniel A. Sampaio, Teresa Albuquerque, Luís Vieira, Vera Manageiro, Manuela Caniça

**Affiliations:** ^1^National Reference Laboratory of Antibiotic Resistances and Healthcare Associated Infections, Department of Infectious Diseases, National Institute of Health Doutor Ricardo JorgeLisbon, Portugal; ^2^Centre for the Studies of Animal Science, Institute of Agrarian and Agri-Food Sciences and Technologies, Oporto UniversityOporto, Portugal; ^3^Microbiology and Mycology Laboratory, Instituto Nacional de Investigação Agrária e VeterináriaLisbon, Portugal; ^4^Innovation and Technology Unit, Human Genetics Department, National Institute of Health Doutor Ricardo JorgeLisbon, Portugal

**Keywords:** *qnrD1*, plasmid, multidrug resistance, *Morganella morganii*, WGS

## Abstract

*Morganella morganii* is a commensal bacterium and opportunistic pathogen often present in the gut of humans and animals. We report the 4.3 Mbp draft genome sequence of a *M. morganii* isolated in association with an *Escherichia coli* from broilers in Portugal that showed macroscopic lesions consistent with colisepticemia. The analysis of the genome matched the multidrug resistance phenotype and enabled the identification of several clinically important and potentially mobile acquired antibiotic resistance genes, including the plasmid-mediated quinolone resistance determinant *qnrD1*. Mobile genetic elements, prophages, and pathogenicity factors were also detected, improving our understanding toward this human and animal opportunistic pathogen.

## Introduction

The Gram negative *Morganella morganii* belongs to the tribe *Proteeae* of the family *Enterobacteriaceae* (O’Hara et al., 2000). This species, along with other elements of *Proteus* and *Providencia* genera can be found in the normal flora of humans, reptiles and in the wider environment (O’Hara et al., 2000; Lee and Liu, 2006; Dipineto et al., 2014). However, *M. morganii* isolates also constitute clinically relevant opportunistic pathogens, which can cause a variety of infections. Nosocomial outbreaks have been reported, suggesting that infections caused by *M. morganii* can lead to major clinical problems, such as wounds, urinary tract infections and septicemia (Nicolle, 2001; Tsanaktsidis et al., 2003; Falagas et al., 2006; Lee and Liu, 2006; Lin et al., 2015).

This bacterium has also been associated with infections in animals and with human animal bite wound infections, which suggests that *M. morganii* may also cause zoonotic infectious diseases (Ono et al., 2001; Choi et al., 2002; Abrahamian and Goldstein, 2011; Zhao et al., 2012; Di Ianni et al., 2015).

Several factors can affect the progression and severity of an infection. The presence of pathogenicity determinants is essential to the success of *M. morganii* in any environment, particularly in food animal farms, where the pressure caused by antibiotic treatments and the lack of prophylactic measures to avoid the spread of infectious diseases are usually noteworthy (Chen et al., 2012; Lin et al., 2015). It is globally accepted that horizontal gene transfer plays an important role in the dissemination of antibiotic resistance genes and pathogenicity factors (Huddleston, 2014). Considering that *M. morganii* may share the habitat with other clinically relevant pathogens, the investigation of any multidrug resistant isolate recovered from poultry is an important assignment.

Resistance to quinolones and fluoroquinolones has been increasingly reported among human and veterinary isolates, very likely as a consequence of the great usage of those antibiotics (Tamang et al., 2011). The *qnrD* gene, now denominated *qnrD1* due to the report of a second variant of the gene (Abgottspon et al., 2014), is a relatively uncommon antibiotic resistance gene, which has been described in members of the *Proteeae* family from different origins (Mazzariol et al., 2012; Zhang et al., 2013; Nasri Yaiche et al., 2014). This plasmid-mediated quinolone resistance (PMQR) determinant encodes a protein that protects DNA gyrases and topoisomerases from quinolone inhibition (Cavaco et al., 2009; Jacoby et al., 2014). Carriage of PMQR-encoding genes frequently confers modest increases to the minimum inhibitory concentrations (MIC) of fluoroquinolones (Poirel et al., 2012). Current studies have identified the environment, particularly animals and aquatic habitats, as a reservoir of PMQR genes (Poirel et al., 2012).

The aim of this study was to investigate the molecular background sustaining the multidrug resistance and pathogenicity of a *M. morganii* isolate. In this study, we report the antibiotic susceptibility and the draft genome sequence of a *qnrD1*-harboring avian isolate. The data gathered from bioinformatics analysis may improve our understanding toward this opportunistic pathogen.

## Materials and Methods

### Bacterial Isolation, Antibiotic Susceptibility, and Molecular Characterization

*Morganella morganii* INSRALV892a was recovered in association with *E. coli* INSRALV892b in 2012 from a 13-days old broiler, recovered from a poultry industrial unit in Portugal. Samples consisted of organs (macerates of liver and spleen) collected during post-mortem examination that were submitted for bacteriological analysis. During post-mortem examination, the birds showed macroscopic lesions consistent with colisepticemia: aerosaculitis, acute enteritis, perihepatitis, and fibrinous peritonitis. Suspected *Enterobacteriaceae* colonies obtained in MacConkey agar plates were isolated in non selective media and identification was performed using API 20E strips (BioMérieux, Marcy-l’Étoile).

Minimum inhibitory concentrations were determined for both isolates by agar dilution method to ten antibiotics: ampicillin, cefotaxime, ceftazidime, meropenem, ciprofloxacin, gentamicin, chloramphenicol, trimethoprim, colistin, and tigecycline. To assess non-susceptibility, interpretation of results was performed according to the clinical breakpoints of the European Committee on Antimicrobial Susceptibility Testing (EUCAST)^1^.

Plasmid-mediated quinolone resistance [QnrA, QnrB, QnrC, QnrD, QnrS, QepA, OqxAB, and Aac(6′)-Ib-cr]-, β-lactamase (TEM, SHV, OXA-G1, and CTX-M)-, and integrase (class 1, 2, and 3)-encoding genes were identified by PCR and confirmed by sequencing using DNA of both isolates, as previously described (Clemente et al., 2013).

The transference ability of specific antibiotic resistance genes from *M. morganii* INSRALV892a and *E. coli* INSRALV892b was assessed by broth mating out assays using *E. coli* J53 NaN3^R^ as recipient strain, as described elsewhere (Jones-Dias et al., 2016). Resistant *E. coli* J53 transconjugants were then selected on MacConkey agar plates containing amoxicillin (100 mg/l) or ciprofloxacin (0.06 mg/l) together with sodium azide (200 mg/l), according with the antibiotic susceptibility profile of the donor isolates. To confirm the acquisition of the antibiotic resistance genes, we detected and identified the determinants in the transconjugants, following the methodology described above in this section.

### Genome Sequencing and Analysis

Genomic DNA of *M. morganii* INSRALV892a was extracted using DNeasy Blood and Tissue Kit (Qiagen, Aarhus), and DNA quantification was performed by Qubit Fluorometric Quantitation (Thermo Fisher Scientific, Carlsbad, CA, USA), according to the manufacturer’s instructions. Libraries were prepared from 1 ng of genomic DNA using the Nextera XT DNA Sample Preparation Kit (Illumina, San Diego, CA, USA), also following manufacturer’s instructions. Whole Genome Sequencing (WGS) was performed using 150 bp paired-end reads on a MiSeq (Illumina, San Diego, CA, USA).

Sequence reads were then trimmed and filtered according to quality criteria. Briefly, reads were assembled *de novo* using CLC genomics workbench version 8.5 (Qiagen, Aarhus), which is based on Smith and Waterman algorithm. The raw FASTQ reads were first processed by quality score trimming (quality score limit = 0.05), removing all reads containing more than two ambiguous nucleotides or shorter than 50 bp. Trimmed reads were then *de novo* assembled with automatic bubble, word size and paired distance detection, using mapping mode “map reads back to contigs” (including scaffolding, and minimum contig length of 400 nucleotides). The NCBI prokaryotic genome automatic annotation pipeline (PGAAP) was used for annotation^2^. All *de novo* contigs were BLAST searched against the GenBank’s non-redundant nucleotide collection (nr/nt). PathogenFinder 1.1, ResFinder 2.1, and PlasmidFinder 1.3 were used to estimate the number and type of pathogenicity determinants, antibiotic resistance genes and plasmids, respectively, within the genome (Zankari et al., 2012; Cosentino et al., 2013; Carattoli et al., 2014). PHAST search web tool was used to identify and annotate any prophage sequence present in the draft genome (Zhou et al., 2011). ISsaga semi-automatic annotation system was also applied to detect the presence of insertion sequences (IS) (Varani et al., 2011).

Contigs containing antibiotic resistance genes were searched for identity through blastn^3^ against the nr/nt NCBI database, and further mapped against the closest bacterial plasmids or genomes using CLC Genomics Workbench version 8.5.

### Nucleotide Sequence GenBank Accession Numbers

This Whole Genome Shotgun project has been deposited at DDBJ/EMBL/GenBank under the accession LGYC00000000. The version described in this paper is the LGYC01000000^4^.

## Results and Discussion

*Morganella morganii* INSRALV892a was found to be non-susceptible to ampicillin (>64 mg/L), cefotaxime (>4 mg/L), ceftazidime (2 mg/L), ciprofloxacin (>8 mg/L), chloramphenicol (16 mg/L), gentamicin (>32 mg/L), trimethoprim (>32 mg/L), colistin (>16 mg/L), and tigecycline (0.5 mg/L). However, it is important to highlight that *M. morganii* is intrinsically resistant to colistin, while tigecycline has also been shown to have poor activity against this species^5^. Among the antibiotics tested, the isolate was susceptible only to meropenem (0.125 mg/L). The *E. coli* INSRALV892b was also characterized with regard to antibiotic susceptibility and found to be non-susceptible to ampicillin (>64 mg/L), cefotaxime (>4 mg/L), ceftazidime (2 mg/L) and trimethoprim (>32 mg/L), and susceptible to meropenem (≤0.03 mg/L), ciprofloxacin (0.125 mg/L), chloramphenicol (≤8 mg/L), gentamicin (≤0.5 mg/L), colistin (≤1 mg/L), and tigecycline (0.5 mg/L). The molecular characterization of the isolates showed the presence of *qnrD1* and a class 2 integron in *M. morganii* INSRALV892a, and *bla*_CTX-M-1_ gene flanked by an IS*Ecp1* and *orf477*, as well as a class 1 integron in *E. coli* INSRALV892b. Conjugation experiments only revealed the transference of *bla*_CTX-M-1_ from *E. coli* INSRALV892b to isogenic *E. coli* J53 strain.

The WGS assembly of *M. morganii* INSRALV892a yielded 74 contigs (each >200 bp long and >100-fold coverage), which together comprised 4,267,817 bp, showing a GC content of 50.6%. The largest contig was 523,676 bp long and the N50 statistic, which stands for the minimum contig length of at least 50% of the contigs, was 342,352 bp. The average length of the obtained contigs was 34,190 bp. Among the obtained data, six contigs, ranging from 802 to 8,575 in length and showing a minimum coverage of 117.7-fold, matched plasmid sequences of different species. Overall, the genome sequence comprised 4,116 putative genes, among which 3,950 consisted of protein encoding sequences.

*In silico* analysis of the antibiotic resistance genes (90% identity and 40% minimum length) revealed the presence of loci for acquired resistance to aminoglycosides [*aadA1y, aph(3*′*)-Ic*, and *strA-strB*], β-lactam (*bla*_OXA-1_), fluoroquinolones (*qnrD1, aac(6*′*)-Ib-cr*), phenicols (*catA2 and catB3*), rifampicin (Δ*arr*), sulphonamides (*sul2*), trimethoprim (*dfrA1*), tetracycline (*tetY)*, and streptothricin (*sat2*). Non-susceptibility to third generation cephalosporins such as cefotaxime and ceftazidime was not associated to any extended-spectrum β-lactamase, suggesting the involvement of inducible or stably derepressed *M. morganii* chromosomal *ampC* gene, the *bla*_DHA-type_ gene (Harris and Ferguson, 2012).

**FIGURE 1:**
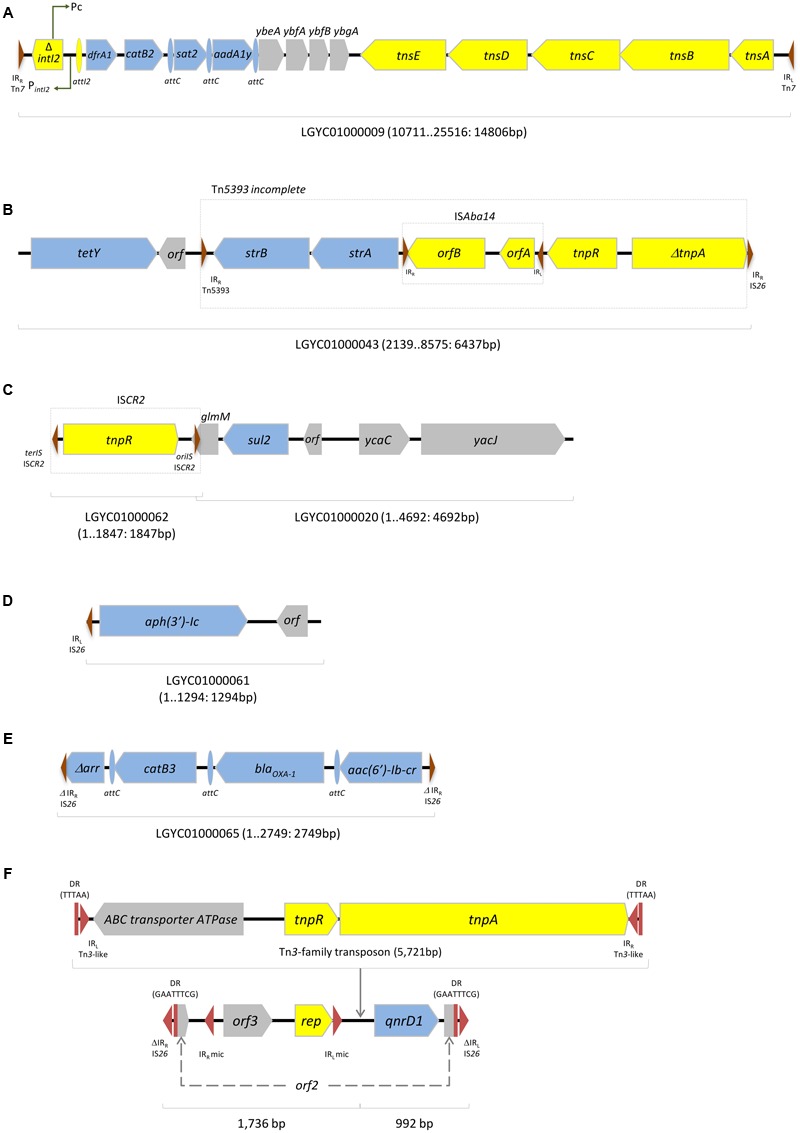
**Examples of contigs containing antibiotic resistance genes in *Morganella morganii* INSRALV892a. (A)** In*2-17* class 2 integron encoding *dfrA1, catB2, sat2*, and *aadA1y*; **(B)** *tetY* and *strA-strB* associated to transposable elements; **(C)** *sul2* flanked upstream by IS*CR2*; **(D)** *aph(3*′*)-Ic* associated to an IS*26* IR; **(E)**Δ*arr, catB3, bla*_OXA-1_, and *aac(6*′*)-Ib-cr* are bracketed by truncated IS*26* IR. **(F)** *qnrD1*-containing region. Blue, antibiotic resistance genes; Yellow, mobile genetic elements; Gray, other genes. Right and left inverted repeats (IR_R_ and IR_L_) and direct repeats (DR) are indicated as red triangles and squares, respectively; mic, mobile insertion cassette.

The *dfrA1, catB2, sat2*, and *aadA1y* genes were enclosed in an In*2-17* class 2 integron that has already been described, for instance, in *Proteus vulgaris* isolates from China (HQ386830) (**Figure 1A**). Genes encoding resistance to tetracycline (*tetY*) and streptomycin (*strA-strB*) were detected in association with each other, and with proteins linked to DNA transfer processes, such as IS*Aba14* and an incomplete Tn*5393* (**Figure 1B**). The sulphonamide resistance gene *sul2* was flanked upstream by a *glmM*-containing region and IS*CR2*, while the downstream region consisted of a chromosomal region typical of *M. morganii* (**Figure 1C**); the *glmM* gene (formerly called *ureC*) encodes a phosphoglucosamine mutase that is considered a housekeeping gene essential for the cell wall synthesis (Tavares et al., 2003). The aminoglycoside resistance gene *aph(3*′*)-Ic* was associated to an IS*26* IR and a unknown *orf* (**Figure 1D**), and the genes Δ*arr, catB3, bla*_OXA-1_, and *aac(6*′*)-Ib-*cr were enclosed together, as gene cassettes of an integron variable region that has been previously found, for instance, in *S. enterica* from livestock (**Figure 1E**). However, in the latter, the array was flanked up and downstream by truncated inverted repeats of IS*26* while no conserved integron regions were found. The genetic regions where antibiotic resistance genes were incorporated were highly similar to other plasmid-borne structures, previously described in different Gram-negative bacteria, suggesting acquisition of resistance determinants through horizontal gene transfer (**Figure 1**).

The *qnrD1* gene was enclosed in an 8,449 bp length contig (LGYC01000051: mean coverage of 183.9-fold and a total read count of 13,382), matching a Col3M plasmid. Indeed, the *qnrD1* gene is frequently located on small non-conjugative plasmids harbored by *Proteeae*, which was corroborated by our conjugation assay (Zhang et al., 2013). Furthermore, the *qnrD1* gene has been located in plasmids showing similarities with a specific *Providencia vermicola* plasmid, suggesting that these small non-conjugative plasmids might be the product of recombination between an unknown *qnrD*-bearing region and a native plasmid from *Proteeae*.

This contig accommodated a 2,683 bp sequence showing 99% identity with previously described *qnrD1*-harboring plasmids, such as pGHS09-09a (HQ834473) and pCGS49 (JQ776507), reported in France and China, respectively. The three *qnrD1*-encoding sequences shared a *rep* gene (also reported as *orf4*) and two additional *orfs* (**Figure 1F**) (Guillard et al., 2012; Zhang et al., 2013). Six single nucleotide variants (SNVs) were detected between *qnrD1*-INSRALV892a and the pGHS09-09a plasmid, two within the *rep* gene. Only one SNP was found with relation to pCGS49 in a non-coding region. The *qnrD1* gene was located within a mobile insertion cassette (mic) element bracketed by two inverted repeats, as previously described (Guillard et al., 2014) (**Figure 1F**).

Comparative bioinformatics analyses revealed the disruption of *orf2* caused by the insertion of IS*26* left and right inverted repeats flanking a region containing *orf3* and *rep*, within a mic, followed by *qnrD1*. In fact, this shows that is possible that LGYC01000051 contig could be either a Col3M plasmid missing an IS*26*-flanked region or a *qnrD1*-containing region that has become incorporated into a larger plasmid.

In addition, this region (**Figure 1F**) included three additional open reading frames: besides an ABC transporter-encoding gene perfectly matching a protein from *Aeromonas hydrophila*, this region harbored Tn*3*-like resolvases- and transposase-encoding genes, displaying *E. coli* plasmid pH226B (KX129784) as its best blast hit.

Mobile genetic elements are crucial tools for the acquisition of genetic diversity (Huddleston, 2014). Thus, we decided to search for and characterize the elements detected in the *M. morganii*’s genome. We identified 10 prophage regions, among which six were incomplete and four were intact, comprising 381 prophage-related genes. Intact prophage regions presented between 24.2 and 41.7 Kb and harbored 13–56 coding DNA sequences. The intact phages showing highest scores were assigned to Enterobacteria phage SfV, which is associated with O-antigen modification and serotype conversion in *Shigella flexneri*, and Enterobacteria phage mEp235 that consists of an unclassified Lambda-like virus (Sun et al., 2013). The bioinformatics detection of IS resulted in the identification of seven transposable elements: IS*3*, Tn*3*, IS*L3*, IS*256*, IS*6*, IS*91*, and IS*As1*. Besides the already mentioned Col3M no other typable plasmids were detected within the *M. morganii* genome, according with the PlasmidFinder tool.

Based on the probability scores assigned by PathogenFinder web-server (Cosentino et al., 2013), the isolate has a probability of acting as a human pathogen of 68.9%, which is in line with the opportunistic nature of this species. *M. morganii*’s genome matched 22 pathogenic families and 5 non-pathogenic families. Pathogenic factors showed diversity of functions and hosts, and included, for instance, transposase *insA* from IS*91* of *Salmonella enterica*, transposition protein *tnsE* of the Tn7 transposon of *S. flexneri*, and transcriptional regulator LysR family protein from *S. enterica*.

Multidrug resistant *M. morganii* isolates are rare and normally associated with non invasive nosocomial opportunistic infections in humans (Nicolle, 2001; Falagas et al., 2006). The detection of an avian *M. morganii* isolate harboring multiple and mobile antibiotic resistance genes and pathogenicity factors raises concerns regarding the dissemination of infection in birds and potential risk of zoonotic transmission. Several factors may affect the susceptibility of poultry to bacterial diseases, namely environmental stressors and previous antibiotic treatments, which are crucial to the development of infections involving different *Enterobacteriaceae* (Burkholder et al., 2008). The detection of an avian *M. morganii* isolate harboring multiple and mobile antibiotic resistance genes and pathogenicity factors raises concerns regarding the dissemination of infection in birds and potential risk of zoonotic transmission.

*M. morganii* is a well characterized opportunistic pathogen (Lee and Liu, 2006). However, its detection in poultry flocks, co-habiting the same hosts as other clinically important pathogens, makes it susceptible to the acquisition and donation of pathogenicity factors by horizontal gene transfer (Huddleston, 2014). To the best of our knowledge this report represents the first genome analysis of an isolate from animal origin carrying *qnrD1*. This genome sequence represents a valuable resource for studies on the epidemiology of zoonotic *M. morganii* isolates, and its features may be used as markers for the study of antibiotic resistance.

## Author Contributions

DJ-D designed the study, performed molecular experiments, analyzed the data and wrote the manuscript. LC performed the microbiological experiments and reviewed the manuscript. IM analyzed the data and reviewed the manuscript. TA, acquired laboratory data. DS and LV performed genome sequencing experiments. VM designed the study, analyzed the data and reviewed the manuscript. MC designed the study, reviewed and edited the manuscript. All authors read and approved the final manuscript.

## Conflict of Interest Statement

The authors declare that the research was conducted in the absence of any commercial or financial relationships that could be construed as a potential conflict of interest.

## Footnotes

1 http://www.eucast.org

2 http://www.ncbi.nlm.nih.gov/genome/annotation_prok/

3 http://blast.ncbi.nlm.nih.gov/Blast.cgi

4 http://www.ncbi.nlm.nih.gov/nuccore/LGYC00000000.1

5 http://www.eucast.org
